# Supplementary material for: Saliva and plasma metabolome analysis during the five days before foaling in the mare
Source: PLoS One. 2026 Apr 1;21(4):e0344530. doi: 10.1371/journal.pone.0344530 (PMC13042699; doi:10.1371/journal.pone.0344530)
Supplement: S2 Table — Compounds that are common to the two biofluids are in italic. (DOCX) [file pone.0344530.s002.docx]

**S2 Table: List of metabolites in each chemical class from saliva and plasma. Compounds that are common to the two biofluids are in italic.**

| **Chemical class** | **Saliva** | **Plasma** |
| --- | --- | --- |
| Alcohols and polyols | myo-Inositol; *Methanol* | Ethanol; Isopropyl alcohol; *Methanol*; 2-Hydroxyisobutyrate |
| Alpha hydroxy acids and derivatives | *Lactic acid* | 2-Hydroxybutyric acid ; *Lactic acid* |
| Alpha-keto acids and derivatives | *Pyruvic acid* | *Pyruvic acid* |
| Amines | *Dimethylamine*; Methylamine | *Dimethylamine* |
| Amino acids, peptides, and analogues | Betaine; *Creatine*; *gamma-Aminobutyric acid*; *Glycine*; *L-Tyrosine*; *Phenylalanine*; *L-Alanine*; *Isoleucine*; Ornithine; L-Arginine; *Leucine*; *L-Valine*; *Phosphocreatine*; 5-Aminopentanoic acid | *Creatine*; Dimethylglycine; *gamma-Aminobutyric acid*; *Glycine*; *L-Tyrosine*; *Phenylalanine*; *L-Alanine*; L-Threonine; L-Asparagine; *Isoleucine*; Histidine; Serine; Creatinine; Glutamine; *Leucine*; Methionine; *L-Valine*; *Phosphocreatine* |
| Benzoic acids and derivatives | *Hippuric acid*; Benzoic acid | *Hippuric acid*; Phthalic acid |
| Beta hydroxy acids and derivatives | *3-Hydroxybutyric acid*; Malic acid | *3-Hydroxybutyric acid*; (S)-3-Hydroxyisobutyric acid |
| Carbohydrates and carbohydrate conjugates | *D-Glucose* ; *Glycerol* | *D-Glucose*; *Glycerol*; D-Mannose |
| Carboximidic acids | *Acetamide*; | *Acetamide*; |
| Carboxylic acids | *Acetic acid*; *Formic acid*; *Propionic acid* | *Acetic acid*; *Formic acid*; *Propionic acid*; Isobutyric acid |
| Fatty acids and conjugates | Butyric acid; *2-Hydroxy-3-methylbutanoic acid*; Isocaproic acid; Isovaleric acid; Valeric acid | *2-Hydroxy-3-methylbutanoic acid*; Capric acid; 3-Hydroxyisovaleric acid; Methylsuccinic acid; 2-Hydroxyvaleric acid |
| Short-chain keto acids and derivatives | *Ketoleucine* | *Ketoleucine*; 2-Oxovaleric acid |
| Sulfones | *Dimethyl sulfone* | *Dimethyl sulfone* |
| Tricarboxylic acids and derivatives | Trans-Aconitic acid | Citric acid |
