# Supplementary material for: Saliva and plasma metabolome analysis during the five days before foaling in the mare
Source: PLoS One. 2026 Apr 1;21(4):e0344530. doi: 10.1371/journal.pone.0344530 (PMC13042699; doi:10.1371/journal.pone.0344530)
Supplement: Attachment — Submitted filename: Response_to_Reviewers.doc [file pone.0344530.s004.doc]

Dr. Ghylène GOUDET

PRC, INRAE, 37380 Nouzilly, France

Phone : 33 (0)2 47 42 79 41

e-mail : ghylene.goudet@inrae.fr

Please find enclosed a marked up version and an unmarked version of our revised manuscript entitled “ Saliva and plasma metabolome analysis during the five days before foaling in the mare“ by L. Nadal-Desbarats, C. Dupuy, F. Montigny, P.S. Bertevello, C. Douet, A. Gesbert, F. Reigner, G. Goudet.

We are looking forward to hearing from you soon.

Sincerely,

Ghylène Goudet

**Journal Requirements:**

1. We have modified the manuscript so that it meets PLOS ONE’s style requirements, the modifications have been highlighted.

2. Information regarding the experiments involving animals and details on methods of sacrifice, anesthesia, analgesia, and efforts to alleviate suffering have been included and highlighted in the Methods section lines 115 to 151, and in the Ethics statement section lines 452 to 464.

3. We have included the requested information about NMR spectroscopy data:

1) In the methods section (line 172 to 179 in the revised manuscript), we have already mentioned the source of the NMR instrument, the magnetic field strength, the nucleus being measured, the solvent, the temperature and the pulse sequence to supress water signal: “^1^H-NMR spectra were acquired at 298 K on an AVANCE III HD 600 MHz system (Bruker Biospin, Karlsruhe, Germany) equipped with a 5 mm TCI cryoprobe with z-gradient. ^1^H-NMR spectra were recorded with a “noesypr1d” pulse sequence with a time domain of 64 K data points, a sweep width of 12 ppm, 64 scans, an acquisition time of 4.56s and a relaxation delay of 20s. A NUS (50%) TOCSY sequence was used on the quality controls for the metabolite identifications of the biological samples. The ^1^H-NMR spectra were phase corrected and imported in NMRProcFlow (DOI: 10.1007/s11306-017-1178-y) for baseline correction and bucketing. Regions with a coefficient of variation across the quality control inferior to 30% were kept for the analysis.”

Lines 169 to 171, the volume of solvent and the standard concentration are already mentioned: “Before NMR analysis, 210 µL of deuterated phosphate buffer (pH = 7.4; containing 3-trimethylsilylpropionic acid (TSP) at 152 µM final concentration) were added to the dried residues and transferred to 3 mm NMR tubes.”

2) The S1-Table resumes, for identified compounds in our biological samples, their chemical shifts in ppm, their multiplicities and their corresponding identifiers according to the Metabolomics Standards Initiative (MSI).

3) Full annotated representative ^1^H-Nuclear Magnetic Resonance spectroscopy spectra of mare saliva and plasma are in Figure 1 (A) and (B). Concerning the chemical structure, the HMDB, PubChem identifiers are mentioned in S1-Table and their SMILES identifiers related to their structure are also resumed in the S1-Table.

The chemical structures were intentionally omitted from the Figure 1 to prevent visual overcrowding.

The NMR data were already deposited on Zenodo Open repository. The data can be uploaded using the following link: <https://doi.org/10.5281/zenodo.17589754>. Line 201 to 202 of the manuscript mentioned: “The experimental data have been deposited with the DOI 10.5281/zenodo.17589754 in the Zenodo repository (https://zenodo.org).”

4. We have expanded the acronym INRAE to ‘Institut National de Recherche pour l’Agriculture, l’Alimentation et l’Environnement’, and we have highlighted the modifications.

5. We have stated the financial disclosure and the role of the funders and highlighted these changes lines 447 to 450:

Financial disclosure

This work was supported by Institut National de Recherche pour l’Agriculture, l’Alimentation et l’Environnement (INRAE) and Institut Français du Cheval et de l’Equitation (IFCE). The funders had no role in study design, data collection and analysis, decision to publish, or preparation of the manuscript.

6. The reviewer comments did not include a recommendation to cite specific previously published works.

7. We have reviewed our reference list, it is complete and correct.

**Response to Reviewers’ comments:**

5. Review comments to the author:

Abstract, line 29: Replace “level” with “concentration” throughout the manuscript

We thank the reviewer to raise this question. However, the authors deliberately adopted the term “level” rather than “concentration”, because although the spectral intensities are expressed relative to a reference standard, they are not corrected for the number of protons contributing to each signal cluster and therefore cannot be interpreted as absolute concentrations. The data presented correspond to relative signal intensities; accordingly, the use of the term “level” is retained.

Discussion, line 370 (373 in the revised manuscript): the sentence has been modified and highlighted.

Lines 155-156 (159 in the revised manuscript): the sentence has been modified and highlighted.

We have reviewed the figures so that they meet the technical requirements.
